# *NECTIN4* Amplification Is Frequent in Solid Tumors and Predicts Enfortumab Vedotin Response in Metastatic Urothelial Cancer

**DOI:** 10.1200/JCO.23.01983

**Published:** 2024-04-24

**Authors:** Niklas Klümper, Ngoc Khanh Tran, Stefanie Zschäbitz, Oliver Hahn, Thomas Büttner, Florian Roghmann, Christian Bolenz, Friedemann Zengerling, Constantin Schwab, Dora Nagy, Marieta Toma, Glen Kristiansen, Hendrik Heers, Philipp Ivanyi, Günter Niegisch, Camilla Marisa Grunewald, Christopher Darr, Arian Farid, Katrin Schlack, Mahmoud Abbas, Can Aydogdu, Jozefina Casuscelli, Theresa Mokry, Michael Mayr, Dora Niedersüß-Beke, Steffen Rausch, Dimo Dietrich, Jonas Saal, Jörg Ellinger, Manuel Ritter, Abdullah Alajati, Christoph Kuppe, Joshua Meeks, Francisco E. Vera Badillo, J. Alberto Nakauma-González, Joost Boormans, Kerstin Junker, Arndt Hartmann, Viktor Grünwald, Michael Hölzel, Markus Eckstein

**Affiliations:** ^1^ Department of Urology and Pediatric Urology, University Hospital Bonn, Bonn, Germany; ^2^ Institute of Experimental Oncology, University Medical Center Bonn (UKB), Bonn, Germany; ^3^ Center for Integrated Oncology Aachen/Bonn/Cologne/Düsseldorf (CIO-ABCD), Bonn, Germany; ^4^ BRIDGE-Consortium Germany e.V., Mannheim, Germany; ^5^ Department of Medical Oncology, National Center for Tumor Disease (NCT), University Hospital, Heidelberg, Germany; ^6^ Department of Urology and Pediatric Urology, Julius Maximilians University Medical Center of Würzburg, Würzburg, Germany; ^7^ Department of Urology, Marien Hospital, Ruhr-University Bochum, Herne, Germany; ^8^ Department of Urology and Pediatric Urology, University Hospital Ulm, University of Ulm, Ulm, Germany; ^9^ Institute of Pathology, University of Heidelberg, Heidelberg, Germany; ^10^ Institute of Pathology, University Hospital Bonn, Bonn, Germany; ^11^ Department of Urology, University Hospital Marburg, Marburg, Germany; ^12^ Department of Hemostaseology, Oncology and Stem Cell Transplantation, Medical University Hannover, Hannover, Germany; ^13^ Department of Urology, University Hospital Düsseldorf, Düsseldorf, Germany; ^14^ Department of Urology, University Hospital Essen, Essen, Germany; ^15^ Department of Urology, University Medical Center Göttingen, Göttingen, Germany; ^16^ Department of Urology, University Hospital Münster, Münster, Germany; ^17^ Department of Pathology, University Hospital Münster, Münster, Germany; ^18^ Department of Urology, University Hospital, Ludwig Maximilian University of Munich, Munich, Germany; ^19^ Department of Diagnostic and Interventional Radiology, Heidelberg University Hospital, Heidelberg, Germany; ^20^ Clinic Ottakring, Institute of Pathology and Microbiology, Wien, Austria; ^21^ Department of Internal Medicine I, Wilhelminenspital, Wien, Austria; ^22^ Department of Urology, Eberhard Karls University, Tübingen, Germany; ^23^ Department of Otorhinolaryngology, University Medical Center Bonn (UKB), Bonn, Germany; ^24^ Medical Clinic III for Oncology, Hematology, Immune-Oncology and Rheumatology, University Medical Center Bonn (UKB), Bonn, Germany; ^25^ Institute of Experimental Medicine and Systems Biology and Division of Nephrology, RWTH Aachen University, Aachen, Germany; ^26^ Department of Urology, Feinberg School of Medicine, Chicago, IL; ^27^ Department of Medical Oncology, Queen's University, Kingston, Ontario, Canada; ^28^ Department of Urology, Erasmus MC Cancer Institute, University Medical Center Rotterdam, Rotterdam, the Netherlands; ^29^ Department of Urology and Pediatric Urology, Saarland University, Homburg, Germany; ^30^ Institute of Pathology, University Hospital Erlangen, Friedrich-Alexander-Universität Erlangen-Nürnberg (FAU), Erlangen, Germany; ^31^ Comprehensive Cancer Center EMN, University Hospital Erlangen, Friedrich-Alexander-Universität Erlangen-Nürnberg, Erlangen, Germany; ^32^ Bavarian Center for Cancer Research (Bayerisches Zentrum für Krebsforschung, BZKF), Erlangen, Germany; ^33^ Clinic for Internal Medicine (Tumor Research) and Clinic for Urology, Interdisciplinary Genitourinary Oncology at the West-German Cancer Center, Essen University Hospital, Essen, Germany

## Abstract

**PURPOSE:**

The anti-NECTIN4 antibody-drug conjugate enfortumab vedotin (EV) is approved for patients with metastatic urothelial cancer (mUC). However, durable benefit is only achieved in a small, yet uncharacterized patient subset. *NECTIN4* is located on chromosome 1q23.3, and 1q23.3 gains represent frequent copy number variations (CNVs) in urothelial cancer. Here, we aimed to evaluate *NECTIN4* amplifications as a genomic biomarker to predict EV response in patients with mUC.

**MATERIALS AND METHODS:**

We established a *NECTIN4*-specific fluorescence in situ hybridization (FISH) assay to assess the predictive value of *NECTIN4* CNVs in a multicenter EV-treated mUC patient cohort (mUC-EV, n = 108). CNVs were correlated with membranous NECTIN4 protein expression, EV treatment responses, and outcomes. We also assessed the prognostic value of *NECTIN4* CNVs measured in metastatic biopsies of non–EV-treated mUC (mUC-non-EV, n = 103). Furthermore, we queried The Cancer Genome Atlas (TCGA) data sets (10,712 patients across 32 cancer types) for *NECTIN4* CNVs.

**RESULTS:**

*NECTIN4* amplifications are frequent genomic events in muscle-invasive bladder cancer (TCGA bladder cancer data set: approximately 17%) and mUC (approximately 26% in our mUC cohorts). In mUC-EV, *NECTIN4* amplification represents a stable genomic alteration during metastatic progression and associates with enhanced membranous NECTIN4 protein expression. Ninety-six percent (27 of 28) of patients with *NECTIN4* amplifications demonstrated objective responses to EV compared with 32% (24 of 74) in the nonamplified subgroup (*P* < .001). In multivariable Cox analysis adjusted for age, sex, and Bellmunt risk factors, *NECTIN4* amplifications led to a 92% risk reduction for death (hazard ratio, 0.08 [95% CI, 0.02 to 0.34]; *P* < .001). In the mUC-non-EV, *NECTIN4* amplifications were not associated with outcomes. TCGA Pan-Cancer analysis demonstrated that *NECTIN4* amplifications occur frequently in other cancers, for example, in 5%-10% of breast and lung cancers.

**CONCLUSION:**

*NECTIN4* amplifications are genomic predictors of EV responses and long-term survival in patients with mUC.

## Teaser

) NECTIN4 amplification predicts response to EV in mUC and it occurs frequently across solid tumors (app. 25% mUC)

## INTRODUCTION

The anti-NECTIN4 antibody-drug conjugate (ADC) enfortumab vedotin (EV) has been approved for previously treated patients with metastatic urothelial cancer (mUC).^1,2^ The combination of EV plus pembrolizumab (EV/P) was recently approved in metastatic, treatment-naïve and cisplatin-ineligible patients with mUC. More recently, in EV-302, this combination proved to be superior to platinum plus gemcitabine and defined a new standard of care in the first-line setting.^3-6^

**CONTEXT**

**Key Objective**
Can NECTIN4 amplifications be used as a genomic biomarker to predict the response to the anti-NECTIN4 antibody-drug conjugate (ADC) enfortumab vedotin (EV) in patients with metastatic urothelial cancer (mUC)?
**Knowledge Generated**
NECTIN4 amplifications were found to be frequent genomic events in mUC, occurring in approximately 25% of cases. In the EV-treated mUC patient cohort, 96% of patients with NECTIN4 amplifications showed objective responses to EV compared with 32% in the nonamplified subgroup. The frequent occurrence of NECTIN4 amplifications in various cancer types, for example, lung and breast cancers, indicates that this biomarker holds promise for tumor-agnostic clinical development of NECTIN4-targeted ADC.
**Relevance *(M.A. Carducci)***
This hypothesis generating study requires prospective evaluation as a predictive genomic biomarker for EV responses. Given the target of EV as an anti-NECTIN4 ADC, the results are highly plausible and may represent strong classifier for treatment response and improved clinical outcomes.*
  *Relevance section written by *JCO* Associate Editor Michael A. Carducci, MD, FACP, FASCO.

EV is currently administered in an all-comer setting without rational biomarker-based patient selection although there is evidence that its target NECTIN4 is heterogeneously expressed in urothelial cancer (UC) molecular subtypes.^7-9^ In addition, we recently showed that membranous NECTIN4 expression frequently decreased during metastatic spread and correlates with EV response in patients with mUC.^10^ In light of other effective treatment alternatives such as trophoblast cell surface antigen 2 (TROP2)- or human epidermal growth factor receptor 2 (HER2)–directed ADC or fibroblast growth factor receptor inhibitors, a better understanding of the molecular basis for EV responses is urgently needed to improve the rational use of this effective drug for patients with mUC^11-15^ and to optimize its ongoing clinical development in earlier UC stages and other solid tumors.^16-19^

The relationship between copy number variation (CNV), mRNA, and protein expression has been known for decades. As a prime example, anti–HER2-targeted therapy conquered modern oncologic therapy of certain breast cancer subtypes and subsequently other entities in an unprecedented success story. The HER2-targeted ADC trastuzumab deruxtecan (T-DXd) proved to be effective in various HER2-expressing solid cancers, also mUC, with a close correlation with expression status.^20,21^ However, HER2-directed therapy is guided solely on the basis of biomarker testing that aims to identify HER2-overexpressing/*ERBB2*-amplified tumors. Unlike in this setting, anti–NECTIN4 EV, whose therapeutic efficacy has been shown to depend on the expression of its target,^7,10^ is applied without previous tumor biomarker testing. Similar to HER2, whose expression is strongly linked to CNV of *ERBB2*, previous reports linked *NECTIN4* gene expression to gains/amplifications of 1q23.3—where the *NECTIN4* gene is located—occurring in approximately 15%-20% of mUC^22^ with an enrichment of *NECTIN4* amplifications in luminal molecular subtypes of mUC.^23^ Despite the frequency of *NECTIN4* CNVs in mUC, to date, the link between *NECTIN4* CNVs, membranous NECTIN4 protein expression, and especially the clinical potential of *NECTIN4* CNVs to predict EV responses has not been assessed.

Thus, we here assessed *NECTIN4* CNVs and their association with membranous NECTIN4 protein expression in a multicenter cohort of n = 108 EV-treated patients with mUC and correlated the results with EV responses and outcomes. Furthermore, we confirmed the correlation of *NECTIN4* CNVs, mRNA, and protein expression in a The Cancer Genome Atlas (TCGA) pan-cancer analysis and explored the prevalence of *NECTIN4* CNVs representing a potential tumor-agnostic genomic biomarker to predict EV response in multiple cancer entities.

## MATERIALS AND METHODS

### TCGA Data

CNV (Affymetrix single nucleotide polymorphism [SNP] 6.0 array data), transcriptome sequencing (RNA-Seq_v2, log2-transformed RNA-Seq by expectation maximization [RSEM] normalized values), and reverse phase protein arrays (RPPA, only for TCGA-BRCA) were downloaded via cBioPortal^24^ querying 10,712 samples/patients in a TCGA pan-cancer analysis including 32 studies. For the n = 408 bladder cancers from TCGA (TCGA-BLCA), clinical data (age, sex, outcomes) were downloaded from the University of California, Santa Cruz Xena browser.^25^ The TCGA Network calculated CNVs using GISTIC 2.0, and the following values were assigned: –2 = deep deletion; –1 = shallow deletion; 0 = diploid; 1 = gain; 2 = amplification.

### Multicenter EV-Treated mUC Cohort

We retrospectively reviewed medical records of n = 108 EV-treated patients with mUC. All patients received EV as the standard of care. Treatment response was evaluated according to RECIST v.1.1 by site investigators.^26^ Progression-free survival (PFS) was defined as the time from EV initiation to radiologic or clinical progression or death from any cause. Representative formalin-fixed and paraffin-embedded (FFPE) tissue of the primary tumor (PRIM; transurethral resection of the bladder [TURB], cystectomy, or nephroureterectomy) and/or metastatic (MET) tissue was required for inclusion in our explorative biomarker study. When multiple tissue samples were available (matched PRIM + MET in n = 27), we considered the one closest to EV start for our outcome analyses. The study was approved by the ethical review board of the Friedrich-Alexander-University Erlangen-Nürnberg (approval numbers: 329_16B and 97_18Bc) and the Medical Faculty of the University of Bonn (approval number: 372/21). Our biomarker study conforms to REMARK guidelines.^27^

### Non–EV-Treated mUC Cohort

Whole-genome sequencing (WGS) was previously conducted on fresh-frozen metastatic biopsy samples from 116 patients with mUC.^23^ These patients with mUC were enrolled in clinical trials (ClinicalTrials.gov identifiers: NCT01855477 and NCT02925234) for palliative systemic treatments, with none receiving EV (mUC-non-EV). This patient cohort was already described in detail by Nakauma-González et al.^23^ *NECTIN4* CNVs were assessed using GISTIC 2.0.^28^ Sufficient clinical information on outcomes was available for n = 103 patients.

### *NECTIN4* Fluorescence In Situ Hybridization

The *NECTIN4* fluorescence in situ hybridization (FISH) probe was purchased from Empire Genomics (Catalog No. NECTIN4-20-GR, Empire Genomics, Buffalo, NY). The probe is designed to specifically target and bind to the *NECTIN4* gene (NCBI Gene ID: 81607). The probe consisted of a fluorescently labeled DNA probe that specifically binds to the *NECTIN4* gene. All hybridizations were performed in an accredited specialized laboratory for clinical molecular pathology (accredited according to DIN EN ISO/IEC 17020) using a standard protocol.

The slides were analyzed using a fluorescence microscope equipped with appropriate filter sets to detect the fluorescence signal from *NECTIN4* and *CEN1* probes. Representative tumor areas for formal analysis were chosen by an experienced board-certified pathologist (ME; blinded to patient outcomes), and at least 50 nonoverlapping nuclei per sample were assessed. Green (*NECTIN4*) and red (*CEN1*) signals were manually quantified. The *NECTIN4*/*CEN1* ratio was calculated, and a ratio of ≥2.0 qualified tumors as *NECTIN4*-amplified. Tumors with ratio values <2.0 were considered nonamplified. Furthermore, gene copy changes (≥4 *NECTIN4* gene copies per nucleus) without qualifying for an amplification (*NECTIN4/CEN1* ratio below <2.0) were considered as polysome tumors, and polysomy status was correlated with response to EV.

### NECTIN4 Immunohistochemistry

Immunohistochemical staining of NECTIN4 was performed using a VENTANA BenchMark ULTRA autostainer (Ventana, Oro Valley, AZ), as previously described.^10^ The samples were categorized as negative (H-score, 0-14), weak (H-score, 15-99), moderate (H-score, 100-199), or strong (H-score, 200-300), as described previously.^10,29^

### SNP Array

DNA from the cryopreserved tumor specimen was isolated using the AllPrep DNA/RNA Micro Kit (#80284, Qiagen, Hilden, Germany) following the manufacturer’s instructions. Infinium Global Screening Array-24 v3.0 Kit (Illumina, San Diego, CA) was used according to the manufacturer’s protocol for the detection of *NECTIN4* CNVs. Data were analyzed using GenomeStudio version 2.0.5 (Illumina) with cnvPartition CNV Analysis Plugin version 3.2.0 to identify CNV regions and estimate CNV values. CNV values of higher than two were considered as amplification.

### Statistical Analysis

Statistical analysis was performed using R (Version 4.3.0), R Studio (Version 2023.03.1 + 446), and GraphPad Prism (Version 9.4.0).

*NECTIN4* CNV was correlated with NECTIN4 mRNA (log2-transformed RSEM-normalized values) and membranous protein expression (H-score). Nonparametric Mann-Whitney test was used to compare two groups. For comparisons involving multiple groups, the nonparametric Kruskal-Wallis test was used.

The predictive value of *NECTIN4* amplification for response to EV was assessed by comparing best overall response (BOR), progression-free survival (PFS), and overall survival (OS) between *NECTIN4*-amplified and nonamplified tumors.

To evaluate the survival after the start of EV treatment, univariable Kaplan-Meier regressions were performed, and significance was determined using the log-rank test. Multivariate Cox regression analyses were conducted to compare the prognostic value of *NECTIN4* CNV with baseline patient characteristics (age, sex) and the Bellmunt risk factors (Eastern Cooperative Oncology Group >0, hemoglobin level <10 g/dL, and the presence of liver metastasis) ^30^ in relation to PFS and OS after EV initiation.

All *P* values were calculated as two-sided, and a significance level of *P* < .05 was used to determine statistical significance.

## RESULTS

### *NECTIN4* Amplifications Predict Responses and Favorable Outcomes to EV in mUC

**FIG 1.**
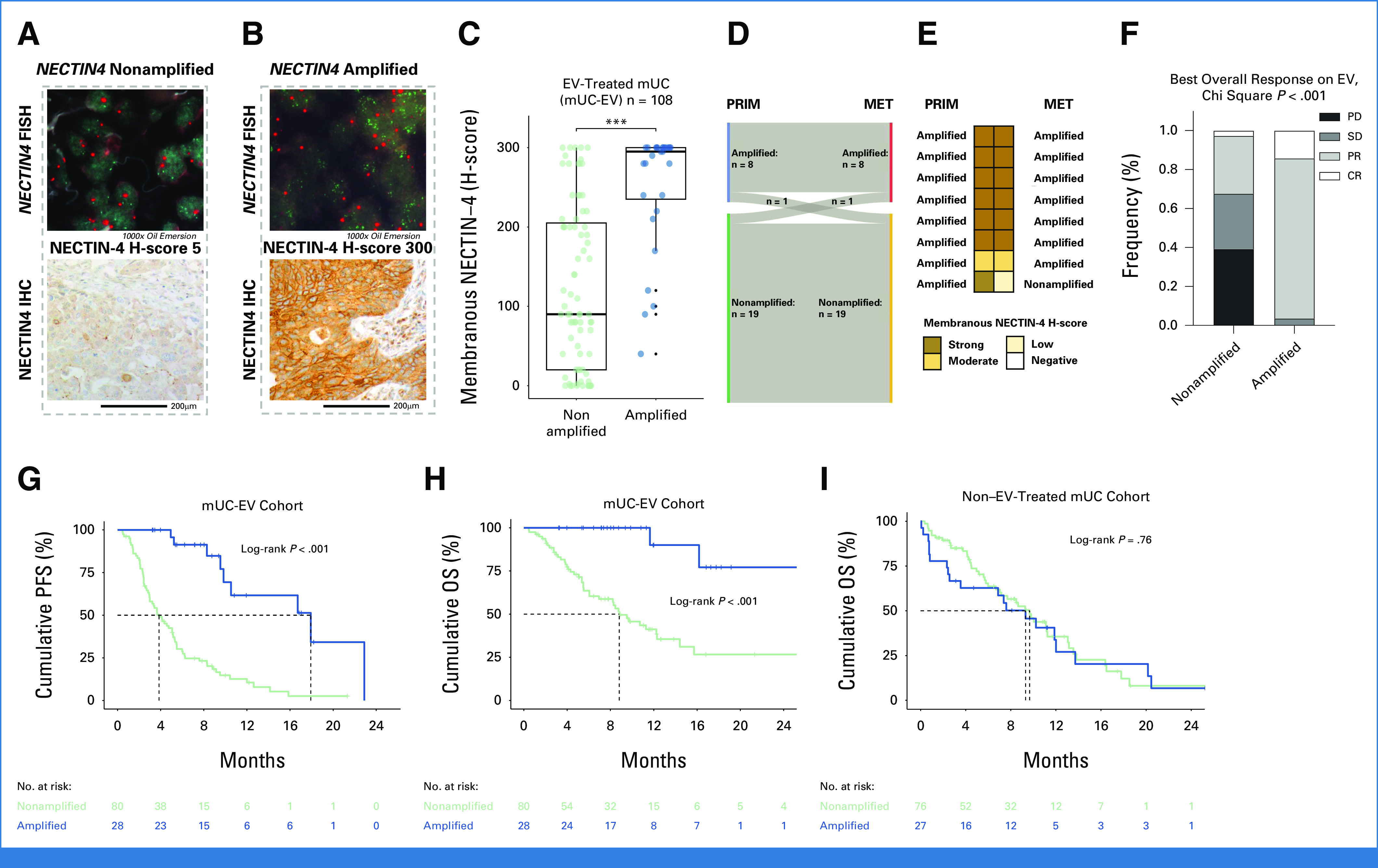
*NECTIN4* amplification predicts EV response in mUC. (A and B) *NECTIN4* FISH image (green signals = *NECTIN4*; red signals = centromere 1, 1,000× oil immersion) and (A) corresponding immunohistochemical NECTIN4 staining on *NECTIN4* nonamplified and (B) *NECTIN4*-amplified urothelial cancers. The gray dashed box demonstrates the two patient cases. (C) Membranous NECTIN4 expression is significantly associated with FISH-detected *NECTIN4* amplification in our EV-treated UC cohort (mUC-EV). Statistical significance (***P < .001) was determined using the Mann-Whitney *U* test. (D) Sankey plot of *NECTIN4* amplification status in the 27 matched PRIM and MET samples. (E) Evolution of membranous NECTIN4 expression during metastatic spread in the eight *NECTIN4*-amplified PRIMs. (F) BOR on the mUC-EV cohort on the basis of *NECTIN4* copy number status; BOR was available for n = 65 patients. *NECTIN4* amplification status is associated with both prolonged (G) PFS and (H) OS since EV therapy start compared with nonamplified tumors. (I) NECTIN4 amplification is not associated with OS in non–EV-treated mUC. The log-rank *P* value is shown. The dashed lines demonstrate median PFS and OS when reached. BOR, best overall response; EV, enfortumab vedotin; FISH, fluorescence in situ hybridization; OS, overall survival; MET, metastatic; PFS, progression-free survival.

**FIG A1.**
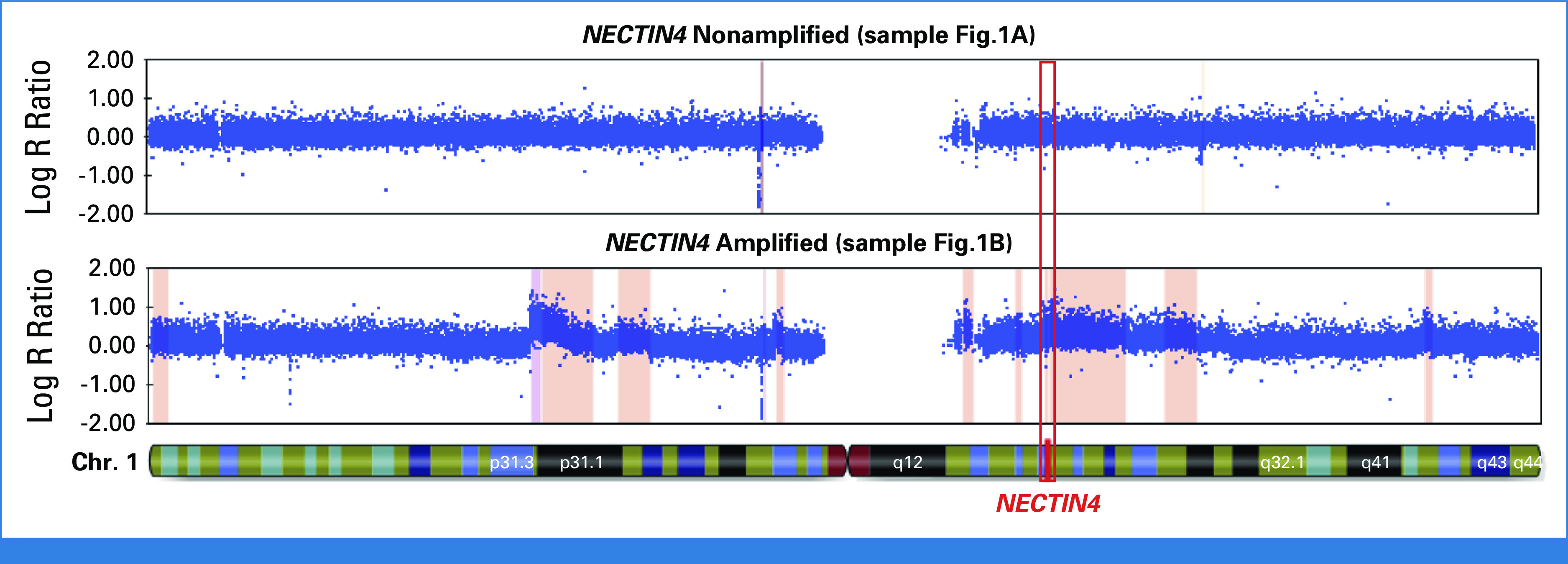
Illustration of copy number variation profiles derived by Illumina SNP arrays. Upper panel: *NECTIN4* nonamplified tumor profile; lower panel: *NECTIN4* amplified tumor profile. The *NECTIN4* gene location on Chr. 1 shows higher copy numbers in the amplified tumors. Chr.1, chromosome 1; SNP, single nucleotide polymorphisms.

We first established a *NECTIN4* FISH assay to examine *NECTIN4* CNVs. FISH images and corresponding IHC stainings for a *NECTIN4* nonamplified UC lacking membranous NECTIN4 expression and a *NECTIN4*-amplified UC that demonstrates pronounced membranous NECTIN4 expression, respectively, are illustrated in Figures 1A and 1B. The *NECTIN4* CNVs in these samples were validated using a SNP assay, which confirms the accuracy and specificity of our *NECTIN4* FISH assay (Appendix Fig A1, online only). Next, we used FISH to determine *NECTIN4* CNV in our multicenter EV-treated mUC cohort (mUC-EV, n = 108). Twenty-eight of 108 samples (26%) showed *NECTIN4* amplifications (*NECTIN4/CEN1* ratio ≥2.0), consistent with amplification frequencies observed in the non–EV-treated metastatic biopsy mUC cohort (mUC-non-EV, 26%, 27 of 103). Regarding baseline characteristics, 25 of 28 patients with *NECTIN4* amplifications were male (*P* = .043) and tended to be older (*P* = .20; Appendix Table A1). In the mUC-non-EV cohort (ClinicalTrials.gov identifiers: NCT01855477 and NCT02925234), 27 of 27 patients with NECTIN4 amplification were male (*P* = .001), and again, there was a nonsignificant trend toward a higher frequency of *NECTIN4* amplification in older patients with mUC (*P* = .069; Appendix Table A2). In TCGA-BLCA, there was a significant correlation between *NECTIN4* amplification and older age (*P* = .013), and there was a nonsignificant trend toward higher amplification frequency in males (*P* = .15; Appendix Table A3). Next, we evaluated whether *NECTIN4* CNVs correlated with membranous NECTIN4 protein expression, the prerequisite for EV binding, known to be correlated with EV response.^10^ *NECTIN4*-amplified tumors demonstrated significantly enhanced membranous NECTIN4 expression (median H-score: 295; IQR, 235-300) compared with *NECTIN4* nonamplified tumors (median H-score, 90; IQR, 20-205; Fig 1C). In 27 matched primary (PRIM) and corresponding metastatic (MET) tumor tissues, *NECTIN4* CNV was stable in 93% (25 of 27). Of eight *NECTIN4*-amplified PRIM with available matched MET, only one tumor lost *NECTIN4* amplification during metastasis (Fig 1D). Membranous NECTIN4 expression of *NECTIN4*-amplified PRIM (median H-score, 290; range, 170-300) remained high in the corresponding MET (median H-score, 280; range, 20-300), except for the primary tumor, which lost its *NECTIN4* amplification (Fig 1E). In only 1 of 27 matched PRIM and MET pairs, *NECTIN4* amplification was exclusive in the metastatic sample (Fig 1D).

**FIG A2.**
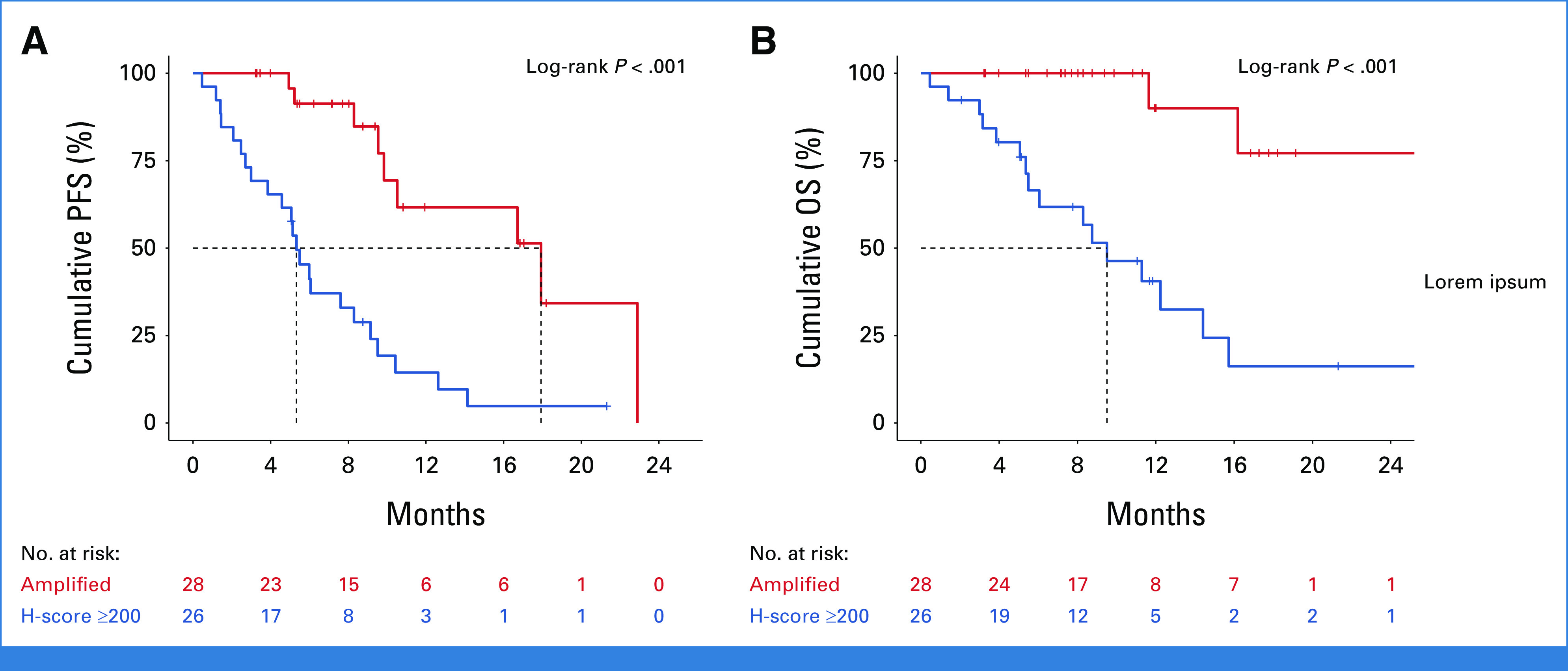
PFS (A) and OS (B) upon initiation of EV treatment stratified by presence of *NECTIN4* gene amplification versus high membranous NECTIN4 protein expression without *NECTIN4* gene amplification. EV, enfortumab vedotin; OS, overall survival; PFS, progression-free survival.

A total of 96% (27 of 28) patients with *NECTIN4* amplification demonstrated an objective response (82%; partial response [PR] and 14% complete response [CR], one patient with stable disease [SD]) as BOR compared with 32% (including 3% with CR) of the *NECTIN4* nonamplified tumors (Chi square *P* < .001; Fig 1F). *NECTIN4* amplifications associated with prolonged PFS (Fig 1G) and OS (Fig 1H), with 90% 12-month survival rate and median OS not reached (95% CI, NR to NR) compared with 41% 12-month survival and a median OS of 8.8 months (95% CI, 6.1 to 14) for *NECTIN4* nonamplified tumors. In multivariable Cox regression coadjusted for age, sex, and Bellmunt risk factors, *NECTIN4* amplification status led to a 92% risk reduction for death compared with *NECTIN4* nonamplified tumors (hazard ratio, 0.08 [95% CI, 0.02 to 0.34], *P* < .001; Table 1). In addition, *NECTIN4* amplification was associated with prolonged PFS and OS compared with the patient subgroup of nonamplified tumors with strong membranous NECTIN4 expression (H-score ≥200; Appendix Fig A2). Furthermore, we explored whether polysome gene copy changes per nucleus (copy number ≥4.0) without qualifying for an amplification (*NECTIN4/CEN1* ratio below <2.0) correlated with EV response and found that five of eight polysome tumors demonstrated an PR/CR or SD with disease control >6 months.

**TABLE 1.** Multivariable Cox Regression Analyses in the Multicenter Enfortumab Vedotin Cohort

Characteristic	PFS				OS			
	No.	HR	95% CI	*P*	No.	HR	95% CI	*P*
*NECTIN4* CNV status				**<.001**				**<.001**
Nonamplified	80	—	—		80	—	—	
Amplified	28	0.14	0.06 to 0.30		28	0.08	0.02 to 0.34	
Age, years				.25				.71
<75	86	—	—		86	—	—	
≥75	22	0.70	0.38 to 1.30		22	1.16	0.54 to 2.47	
Sex				.28				.58
Male	81	—	—		81	—	—	
Female	27	1.36	0.79 to 2.34		27	0.83	0.41 to 1.65	
Liver metastasis				.31				**.023**
No	76	—	—		76	—	—	
Yes	32	0.77	0.46 to 1.29		32	2.06	1.11 to 3.82	
ECOG				.090				**<.001**
0	36	—	—		36	—	—	
≥1	72	1.53	0.92 to 2.55		72	4.84	2.01 to 11.7	
Hemoglobin, g/dL				.54				.16
≥10	104	—	—		104	—	—	
<10	4	0.71	0.23 to 2.19		4	0.29	0.04 to 2.24	

NOTE. Significant *P* values are highlighted in bold.

Abbreviations: CNV, copy number variation; ECOG, Eastern Cooperative Oncology Group; HR, hazard ratio; OS, overall survival; PFS, progression-free survival.

**FIG A3.**
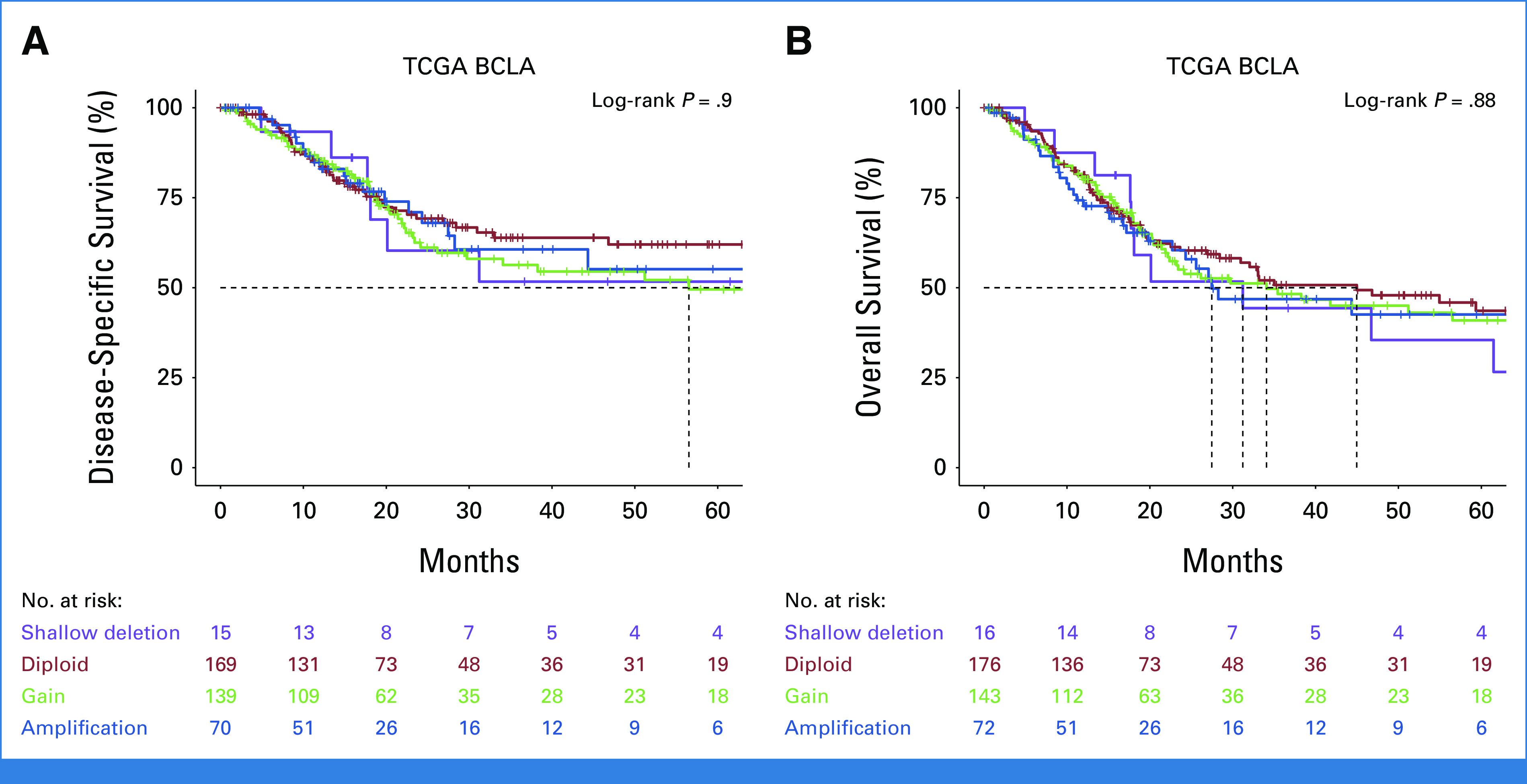
(A) Disease-specific survival of n = 393 patients of the TCGA-BLCA cohort stratified by copy number alterations of *NECTIN4* (data were missing for 14 patients). (B) Overall survival of n = 407 patients of the TCGA-BLCA cohort stratified by copy number alterations of *NECTIN4*. TCGA-BLCA, The Cancer Genome Atlas-bladder cancer.

To rule out a prognostic bias of *NECTIN4* CNVs, we assessed their prognostic impact in non–EV-treated UC patient cohorts. In the mUC-non-EV cohort, *NECTIN4* amplifications were assessed via whole-genome DNA sequencing in 103 metastatic biopsy samples obtained before palliative systemic treatment. In this cohort, *NECTIN4* amplifications were found in 26% of tumors and were not associated with OS (Fig 1I). In the TCGA-BLCA cohort of muscle-invasive bladder cancer, *NECTIN4* amplifications were also not associated with disease-specific survival and OS (Appendix Fig A3A and A3B).

### *NECTIN4* Amplification Occurs Frequently Across Entities

**FIG 2.**
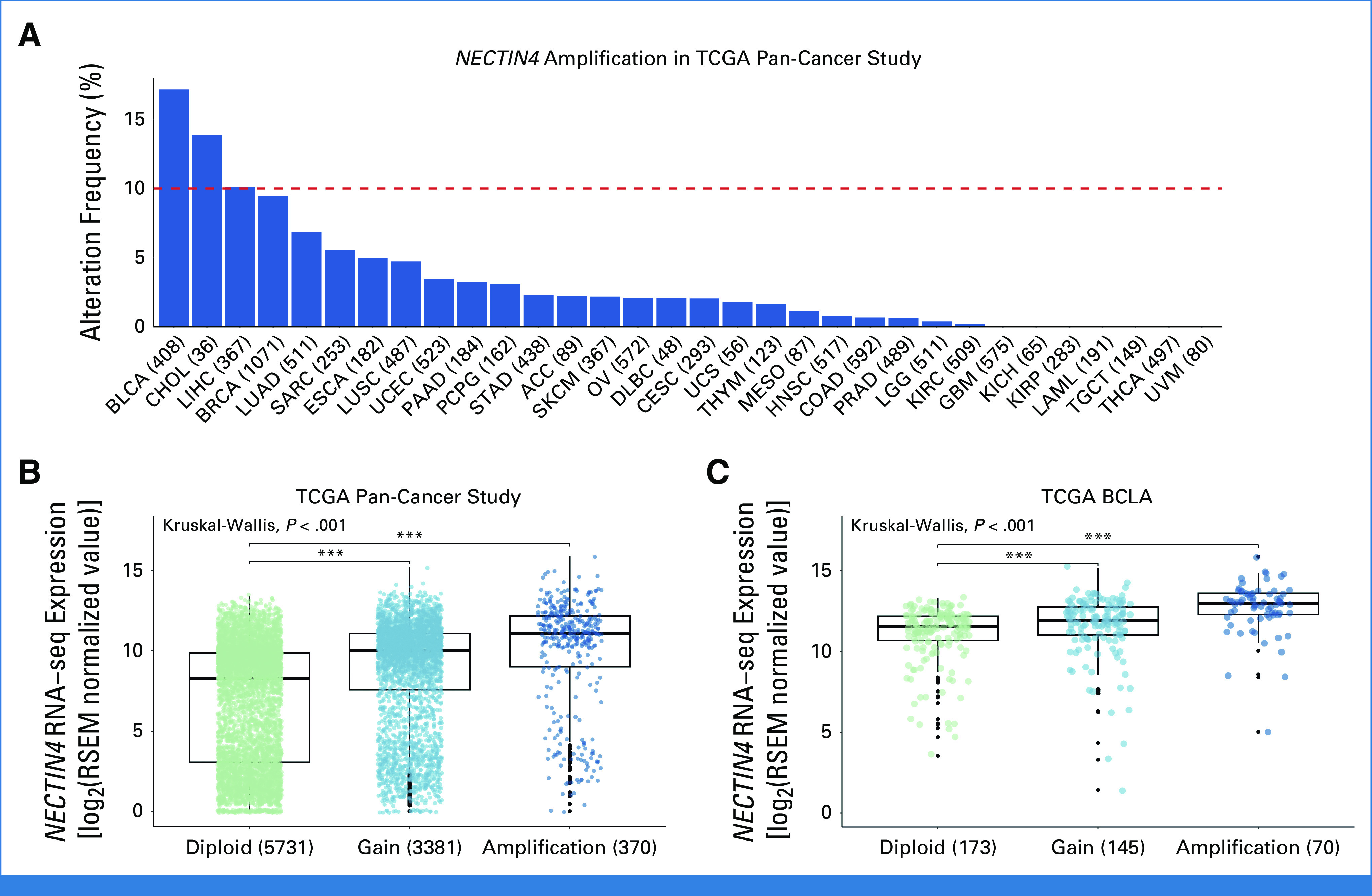
*NECTIN4* amplifications occur frequently across solid tumors. (A) The frequency of *NECTIN4* amplifications are depicted for 32 studies consisting of 10,712 samples/patients, with BLCA presenting the highest prevalence (17%). Positive correlation was observed between *NECTIN4*′ copy number variation and mRNA level in both (B) Pan-Cancer Study and (C) TCGA-BLCA. Standard TCGA study abbreviations were used.^31^ BLCA, Bladder Urothelial Carcinoma; TCGA, The Cancer Genome Atlas.

**FIG A4.**
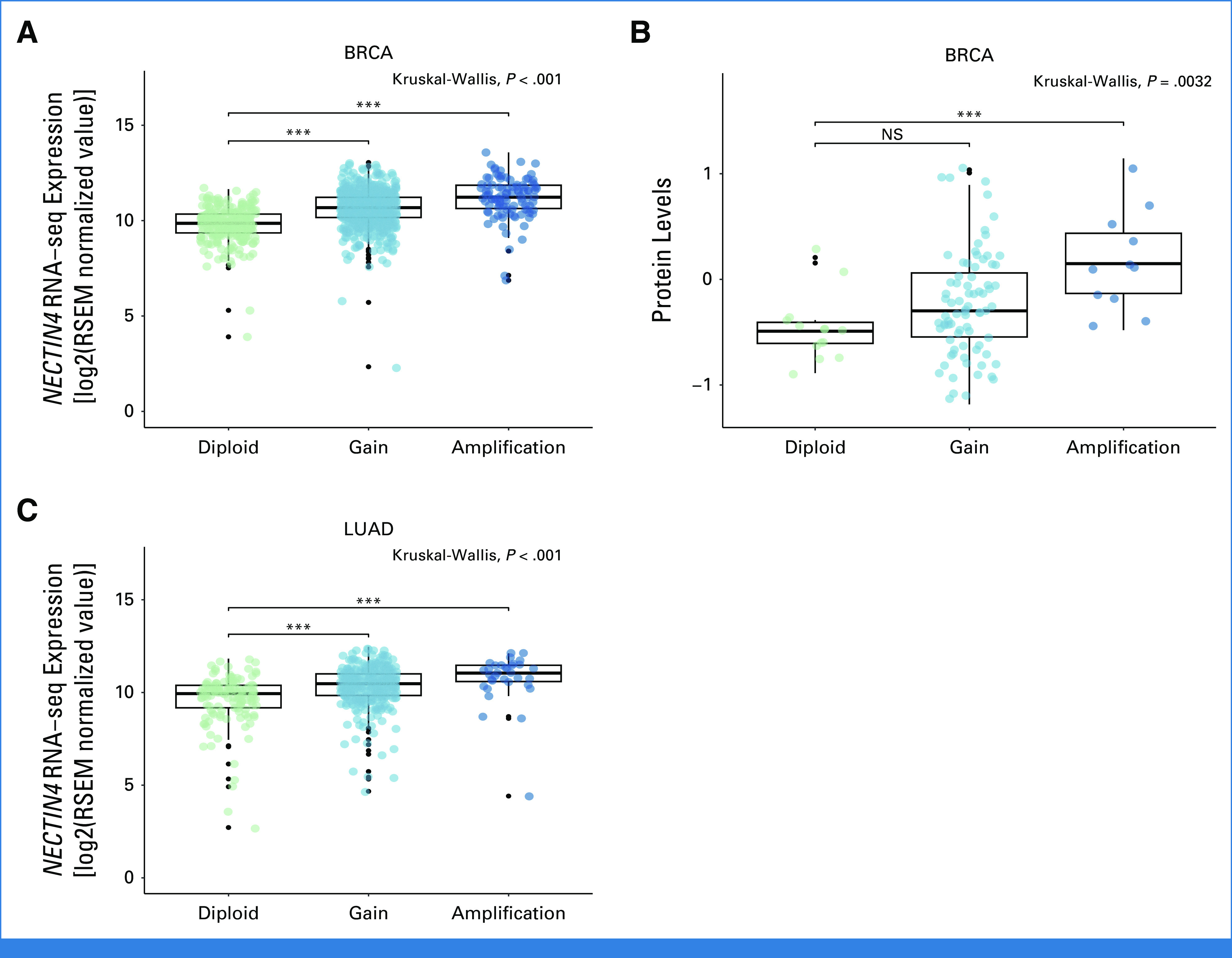
(A) *NECTIN4* mRNA expression (log2 normalized RSEM values) in TCGA-BRCA cohort stratified by *NECTIN4* copy number alterations. (B) NECTIN4 protein expression levels (Z-score scaled results from RPPA) in TCGA-BRCA cohort stratified by *NECTIN4* copy number alterations. (C) *NECTIN4* mRNA expression (log2 normalized RSEM values) in TCGA-LUAD cohort stratified by *NECTIN4* copy number alterations. NS, not significant; RPPA, reverse-phase protein arrays; TCGA-BRCA, The Cancer Genome Atlas breast cancer; TCGA-LUAD; The Cancer Genome Atlas lung adenocarcinoma.

In the TCGA Pan-Cancer cohort, *NECTIN4* amplifications were observed in 25 of 32 cancer types including various solid entities with *NECTIN4* amplification frequency > 5% (Fig 2A). The highest prevalence of *NECTIN4* amplifications was found in bladder cancer (BLCA, 17%), cholangiocarcinoma (CHOL, 14%), hepatocellular carcinoma (LIHC, 12%), breast cancer (BRCA, 9%), and lung adenocarcinoma (LUAD, 7%). *NECTIN4*-amplified samples or those with gains showed increased *NECTIN4* mRNA levels compared with diploid samples (Fig 2B) on the pan-cancer level. In BLCA, BRCA, and LUAD—where EV is approved or in late-stage clinical development—*NECTIN4* amplifications associated with increased *NECTIN4* mRNA expression (Fig 2C; Appendix Figs A4A and A4C) and higher NECTIN4 protein levels in breast cancer (Appendix Fig A4B).

## DISCUSSION

The identification of biomarkers to predict response to targeted therapies is crucial to improve the management of patients with cancer.^32^ Here, we provide data from a multicenter mUC patient cohort highlighting *NECTIN4* amplifications as genomic biomarkers to predict EV responses and favorable outcomes. Importantly, in the non–EV-treated patients with mUC, *NECTIN4* amplifications have no impact on OS,^33^ suggesting that *NECTIN4* amplifications are neither indicating aggressive nor favorable tumor biology, strengthening its potential value as a pure predictive biomarker.^34^ *NECTIN4* amplification was strongly associated with EV sensitivity (BOR, 96%). However, the response rate of 32% in the nonamplified subgroup is comparable with the expected outcomes (BOR app. 40%) observed in real-world settings and the pivotal phase III EV-301 study.^1,35,36^ With a median OS of 12 months (95% CI, 9.7 to NR) in our mUC-EV cohort, our data confirm the clinical activity of EV in previously treated patients with mUC (eg, EV-301, 12.9 months [95% CI, 10.6 to 15.2]). Therefore, EV again proves to be an effective drug in previously treated mUC also in the nonamplified context.

We recently showed that membranous NECTIN4 protein expression is volatile and often (>50%) decreases during metastatic progression of mUC.^10^ By contrast, 88% of PRIM with *NECTIN4* amplifications retains their *NECTIN4* amplification and subsequently a stable high membranous NECTIN4 protein expression during metastatic progression. This is in line with previous results from the study by Faltas et al^37^ demonstrating that early acquired genomic features including copy number alterations are rather stable during metastatic progression in comparison with parental primary tumors. Thus, treatment decisions for the metastatic stage could be based on *NECTIN4* amplification status in primary tumor material, facilitating implementation into clinical trials. It is worth noting that this consideration does not apply to the assessment of membranous NECTIN4 protein expression, which decreases substantially during metastasis in UC without *NECTIN4* amplifications.^10^ This difference could be explained by the inability of *NECTIN4*-amplified tumors to downregulate membranous expression of NECTIN4 at the transcriptional level. Because downregulation of the target is a known mechanism of resistance to ADCs,^38,39^ this could explain, at least in part, the exceptional and durable clinical efficacy of EV in *NECTIN4*-amplified tumors. Beside considerations of tissue choice for predictive biomarker testing, overcoming hurdles to implement biomarker tests into daily care is a major obstacle for biomarker-guided therapies. In the case of CNV assessment, a broad variety of cytogenetic and molecular techniques are available, including FISH/chromogenic in situ hybridization, SNP microarray, comparative genomic hybridization, multiplex ligation-dependent probe amplification, and sequencing methods like whole-exome or whole-genome sequencing.^40^ Among these options, FISH is the most frequently performed diagnostic assay to assess CNVs in clinical routine.^41^ Moreover, FISH as predictive biomarker assay has been proven to be a highly reproducible, easy-to-implement, fast, and cost-effective method in daily molecular pathology. Thus, we conclude that a *NECTIN4* FISH assay could be quickly integrated into clinical trials and routine molecular pathology/daily patient care.

Other biomarkers were described to be associated with EV response and outcomes: Jindal et al^42^ conducted a comprehensive biomarker analysis within the UNITE study cohort, which comprised 303 patients receiving EV monotherapy with available next-generation sequencing data across 16 US sites. Among these patients, 207 had their tumor mutational burden (TMB) assessed and 146 had their PD-L1 status evaluated. Multivariate analysis revealed that alterations in *ERBB2, KDM6A*, and *PIK3CA* were associated with favorable treatment outcomes on EV. Conversely, patients with low TMB (<10 Mut/Mb) and high PD-L1 (CPS ≥10) exhibited less favorable outcomes on EV.^42^ It is known that alterations in *ERBB2* and *KDM6A* are over-represented in luminal differentiated UC,^43^ which are known to be enriched for *NECTIN4* amplification ^23^ and increased NECTIN4 mRNA and protein expression.^7,44^ Therefore, the prognostic value of these genomic alterations may depend on luminal differentiation and concomitant higher NECTIN4 expression. Consistent with this, the absence of squamous differentiation has been shown to correlate with response to EV.^45^ In addition, the occurrence of skin toxicity after initiation of EV treatment has been reported to be associated with favorable outcomes of EV treatment.^46^ In the context of ADC precision oncology, it is well established from several clinical trials that ADC response correlates with the respective target gene expression, for example, for HER2^14,20,21^ and FOLR1-targeting ADC ^47^; we have demonstrated linear correlation also between membranous NECTIN-4 expression and EV response.^10^ Future biomarker analyses would therefore ideally need to integrate membranous NECTIN4 expression, *NECTIN4* CNV, histomorphology, and further high throughput data to deepen our understanding of EV-responsive tumors.

Rational biomarker-guided therapy selection is urgently required to establish the optimal therapy sequence for patients with (m)UC.^11,13,32,48^ Consideration of *NECTIN4* amplifications as predictive biomarkers could potentially rationalize EV drug development—also at earlier disease stages—by defining the patient subgroup with the highest chance of durable benefit. In this context, a strategic focus on biomarker-guided trials could greatly enhance our understanding of the potential of EV or other anti–NECTIN4-targeted therapies and open new avenues to optimize treatment and improve outcomes in patients with (m)UC.^48,49^

A wide range of surface targets, such as HER2 or TROP2, are present in different types of cancers, and there has been a growing interest to expand the use of ADC beyond specific cancer types in a tumor-agnostic fashion.^16,17,50,51^ Of note, in our TCGA Pan-Cancer analysis, *NECTIN4* amplifications can be found in 5%-10% of breast cancer and non–small cell lung cancer, both tumor types with a high impact on all-cancer mortality, which are currently being evaluated for EV response in the multicohort phase II EV-202 trial (ClinicalTrials.gov identifier: NCT04225117).^19^ Thus, *NECTIN4* CNV may be a valuable predictive biomarker to streamline clinical development of NECTIN4-targeted therapies in tumor entities beyond UC.^52^ The frequent occurrence of *NECTIN4* amplifications across solid cancer types could thus pave the way for basket trial designs studying the efficacy of EV on the basis of *NECTIN4* CNV status in a tumor-agnostic study framework,^16,17^ similar to the phase II DESTINY-PanTumor02 trial which assessed anti-HER2 ADC T-DXd in HER2-expressing solid tumors.^20^

Although our study certainly has important strengths, its main limitation is the use of a retrospectively assembled patient cohort, which consists of both archived primary (TURB, cystectomy or nephroureterectomy) and metastatic tumor specimens with varying ranges between tumor sampling and start of EV treatment. Therefore, our data are hypothesis-generating and prospective confirmation in larger, biomarker-driven trials is mandatory. As the combination of EV/P is the new standard of care in the first-line treatment of mUC, the predictive value of *NECTIN4* amplification in this new treatment setting should be further investigated. In addition, our study does not include correlative data on *NECTIN4* CNVs and responses to EV in other cancer entities, as mUC is the only approved standard-of-care setting for EV to date.

In conclusion, our study suggests that *NECTIN4* amplification is a simple, valuable, and easy-to-implement predictive biomarker for EV in patients with mUC. The frequent occurrence of *NECTIN4* amplifications in other cancer types suggests that this biomarker is a promising candidate with broader applicability for clinical development of NECTIN4-targeted ADCs in a tumor-agnostic context.

## Data Availability

The results are in part based upon publicly available data generated by the TCGA Research Network: https://www.cancer.gov/tcga. Further data that support the findings of this study are available from the corresponding author upon reasonable request.

## APPENDIX

**TABLE A1.** Baseline Characteristics of the mUC-EV Cohort

Characteristic	Nonamplified (n = 80)	Amplified (n = 28)	*P* ^a^
Sex, No. (%)			**.043**
Male	56 (70)	25 (89)	
Female	24 (30)	3 (11)	
Age, years			.2
Median (IQR)	68, (58-73)	69, (61-75)	
Range	33-89	55-89	
ECOG, No. (%)			.9
0	26 (33)	10 (36)	
1	43 (54)	14 (50)	
≥2	10 (13)	4 (14)	
Hemoglobin, g/dL, No. (%)			.6
≥10	76 (95)	28 (100)	
<10	4 (5.0)	0 (0)	
Liver metastases, No. (%)			**.011**
No	51 (64)	25 (89)	
Yes	29 (36)	3 (11)	

NOTE. Significant *P* values are highlighted in bold.

Abbreviations: ECOG, Eastern Cooperative Oncology Group; mUC-EV, metastatic urothelial cancer-enfortumab vedotin.

^a^ Pearson's chi-squared test; Wilcoxon rank-sum test; Fisher's exact test.

**TABLE A2.** Baseline Characteristics of mUC-Non-EV

Characteristic	Nonamplified (n = 76)	Amplified (n = 27)	*P* ^a^
Sex, No. (%)			**.001**
Male	53 (70)	27 (100)	
Female	23 (30)	0 (0)	
Age, years			.069
Median (IQR)	67 (56-72)	71 (62-75)	
Range	39-82	25-85	

NOTE. Significant *P* values are highlighted in bold.

Abbreviation: mUC-Non-EV, metastatic urothelial cancer-non enfortumab vedotin.

^a^ Pearson's chi-squared test; Wilcoxon rank-sum test.

**TABLE A3.** Baseline Characteristics of TCGA-BCLA Cohort

Characteristic	Nonamplified, n = 336	Amplified, n = 72	*P* ^a^
Sex, No. (%)			.15
Male	243 (72)	58 (81)	
Female	93 (28)	14 (19)	
Age, years			**.013**
Median (IQR)	68 (60-75)	73 (66-78)	
Range	34-90	44-88	

NOTE. Significant *P* values are highlighted in bold.

Abbreviation: TCGA-BCLA, The Cancer Genome Atlas - Bladder Cancer.

^a^ Pearson's chi-squared test; Wilcoxon rank-sum test.
